# Management of caffeine in wastewater using MOF and perovskite materials: optimization, kinetics, and adsorption isotherm modelling

**DOI:** 10.1007/s40201-024-00904-2

**Published:** 2024-05-20

**Authors:** Amira Essam, Samaa Imam Eldek, Nabila Shehata

**Affiliations:** 1 Environmental Science and Industrial Development Department, Faculty of Postgraduate Studies for Advanced Sciences, Beni-Suef University, Beni-Suef, Egypt; 2 Materials science and Nanotechnology Department, Faculty of Postgraduate Studies for Advanced Sciences, Beni-Suef University, Beni-Suef, Egypt

**Keywords:** Adsorption, Caffeine, MOF, Perovskite, Water treatment

## Abstract

Pharmaceuticals and personal care products (PPCPs) have been increasingly used all over the world and they have been reported on water cycle and cause contamination. Among these pharmaceuticals is caffeine (CAF). In this work, CAF removal from aqueous samples by metal–organic framework (UIO-66) and perovskite (La_0.7_Sr_0.3_FeO_3_) was achieved. Detailed studies on the preparation of MOFs and perovskite oxides compounds have been presented. Extensive characterizations such as X-Ray diffraction (XRD), field emission scanning electron microscope (FESEM), Fourier transform infrared spectra (FT-IR), N_2_ adsorption–desorption isotherms were also carried out to assure proper formation and to better understand the physico-chemical behavior of the synthesized samples before and after adsorption. Batch experiments of CAF adsorption onto both MOFs and perovskite were performed to compare the effectiveness of both materials on the removal competence of the CAF residue at different conditions including the effect of pH, initial concentration, and contact time. It was observed that the adsorption capacity of CAF by MOF increased with increasing acidity. On the other hand, the adsorption capacity of perovskite is stable in pH 4–10. The maximum adsorption capacities of UiO-66 and perovskite toward CAF are high as 62.5 mg g^−1^ and 35.25 mg g^−1^, respectively. Equilibrium isotherms were investigated by numerous models: Langmuir, Freundlich, Temkin, Redlich-Peterson, Sips, Langmuir-Freundlich, Toth, Kahn, Baudu, and Fritz Schlunder. Moreover, the kinetics of the CAF@MOF and CAF@Perovskite systems have been studied by five kinetic models (Pseudo-1st -order (PFO), Pseudo-2nd -order (PSO), Mixed 1st, 2nd-order, Intraparticle diffusion and Avrami). The best model described the adsorption of CAF onto both of MOF and perovskite was the mixed 1st, 2nd-order model. The metal–organic framework and perovskite were applied to quickly extract CAF from water samples successfully. The maximum removal percentage obtained for MOF and perovskite was 0.89% and 0.94% respectively within 30 min contact time which suggests that these materials are considered as promising adsorbents for CAF.

## Introduction

 In recent years, using of pharmaceuticals and personal care products (PPCPs) has been increased all over the world with a special focus in developed countries. Subsequently, trace pharmaceuticals have been reported in the water cycle, including wastewater, surface water and groundwater. There are serious problems with the contamination of water resources, including groundwater with PPCPs [1]. Moreover, PPCPs were found in the tissues of fish and vegetables. These compounds have the capacity to cause sexual problems in aquatic animals as well as genetic defects in organisms [2]. Among these pharmaceuticals, caffeine (CAF, 1,3,7- trimethyl xanthine) is one of the most concerned chemical compounds due to its widely implementation as a drug for therapeutic applications such as some analgesic and bronchodilator drugs [3], and it has been considered as one of the main components of coffee and green tea. International Coffee Organization (ICO) reported that coffee which consumed in the world wide is approximately 1.6 billion cups every day [4]. Moreover, CAF is a naturally occurring alkaloid in approximately 60 plant species. CAF is a small water-soluble molecule having simple structure. It can increase lipolysis rate and enzyme activity. Accordingly, CAF is usually used in cosmetic formulations such as emulsions or hydrogels [3]. The research reported that high amount of CAF can result in restlessness, irritability, nervousness, and fast heartbeat [4]. Thus, it is necessary to remove the contamination of CAF from water. Water and wastewater treatment plants don’t effectively remove PPCPs such as CAF from water. Some water remediation processes such as reverse osmosis and nanofiltration were suggested to remove pharmaceuticals from water. However, using of reverse osmosis and nanofiltration tend to be expensive. Although, other processes such as photodegradation and advanced oxidation processes (AOPs) have been applied to remove PPCPs from water resources, still some challenges toward these methods to be worked efficiently. Researchers widely used adsorption technique in removing contaminants from water owning to its high efficiency, simplicity, cost effectiveness, variety of the adsorbents, and it can be used under mild conditions with low energy consumption [1]. Various materials have been extensively studied as adsorbents to manage CAF in water such as activated carbons [5], biochar [5], ZnO/zeolite pellets [6], layer double hydroxide (LDH) [7], perovskite [8] and metal-organic frameworks )MOFs) [9].

Among these adsorbents, metal-organic frameworks (MOFs) are characteristic with their selectivity [10]. The MOFs are crystalline porous solids containing one-, two- or three dimensional networks of clusters or metal ions held in place by multidentate organic ligands [11]. By changing type of organic ligands and metal cores, many possible MOFs with various properties can be synthesized [12]. Large porosity and tunable surfaces offered by these crystalline structures make them suitable targets in a variety of biological, gas adsorption, chemical catalysis, sensing, molecule separation, and pharmacology [13].

The MOFs are composed of both organic and inorganic moieties [1] and they have been employed as platforms for anchoring catalytic nanoparticles effectively [2]. MOFs have been applied in the removal of some hazardous organics such as ciprofloxacin (CIP), norfloxacin (NOR) and methyl orange (MO) [14], heavy metal ions such as Hg^2+^ [14] and Cr(VI) [15], PPCPs [16], owning to their high specific surface areas and pore volumes. Their geometry and pore size can be modified with a number of post-synthesis or direct functionalization methods and attract much attention compared with other porous materials due to their easy synthesis [9]. MOFs have been investigated for the management of various pharmaceutical residues from water. For example, ZR-MOF(UIO-66) has been modified by chitosan (CSF@UiO-66) to adsorb ketoprofen from aqueous solution and the maximum adsorption capacity reached 209.7 mg g^−1^ at pH 4 [16]. Sulfamethoxazole was removed from water by UiO-66 and UiO-66-BC composites [17]. UIO-66 has been also investigated for the adsorption of theophylline and CAF. The maximum adsorbed amounts of theophylline and CAF onto UIO-66 were 0.451 and 0.392 mg g^−1^, respectively [9] and the best model found for the adsorption of theophylline and CAF onto UIO-66 was Langmuir–Freundlich model with regression coefficients (R^2^) 0.976 and 0.989, respectively. MOFs have been used to manage CAF in water and it was found that there are many factors impact the adsorption of CAF such as pH and temperature [18]. The initial concentration and contact time are also considerable parameters that affect the adsorption process of CAF [19].

On the other hands, perovskite oxides have attracted significant attention due to their eligible physicochemical properties. The semiconductor lanthanum strontium ferrite (LaSrFeO_3_) is one of the most common AFeO_3_ perovskite oxides and it can be excited by visible light irradiation due to its narrow band gap [20]. The synthesis of LaSrFeO_3_ has been achieved by many methods, including mechanochemical solid reaction, solid-state reaction, combustion synthesis and wet chemical co-precipitation [21]. To the best of the author’s knowledge, it is the first study on the management of a pharmaceutical product in wastewater using perovskite as an adsorbent. Also, this study is characteristic with a facile preparation method (one step) of perovskite.

The main aim of this work is to investigate the affinity of MOF and perovskite materials towards CAF as a model PPCP. Hence, detailed studies on the preparation of MOFs and perovskite oxides compounds have been presented. Extensive characterizations including FESEM, FT-IR, N_2_ adsorption–desorption isotherms and XRD analyses, were also carried out to assure proper formation and to better understand the physico-chemical behavior of the synthesized samples. Batch experiments of CAF adsorption onto both MOFs and perovskite were performed to compare the effectiveness of both materials on the removal competence of the CAF residue at different conditions including pH, initial concentration, and contact time followed by adsorption isotherm modeling. Finally, kinetic studies using Pseudo-1st -order (PFO), Pseudo-2nd -order (PSO), mixed 1st, 2nd-order, intraparticle diffusion and Avrami models were investigated for better understanding of the adsorption performance of both materials towards CAF.

## Materials and methods

### Materials

Dimethylformamide (DMF), acetic acid, 1,4-benzenedicarboxylicacid (BDC), ZrOCl_2_ were bought from El-Salam for chemical industries (Egypt), LOBA Chemie (India), Fine-Chem Limited (Mumbai), Sigma-Aldrich (Germany), respectively. Ferric nitrate, lanthanum nitrate, strontium nitrate, citric acid, and ammonia solution were purchased from LobaChemie (Mumbai), Oxford Laboratory (Mumbai), Alpha Chemika (Mumbai), and LobaChemie (India), respectively. All the chemical reagents were used without further purification.

### Methods

#### Synthesis of MOF

1,4-benzenedicarboxylic acid (BDC, 740 mg), ZrOCl_2_ (750 mg), and acetic acid (4 mL) were mixed in DMF (90 mL) using ultra-sonication for 30 min. The solution was introduced to a Teflon-lined stainless-steel autoclave (100 mL) and heated at 120 °C for 24 h. The reaction solution was cooled to room temperature and washed three times using ethanol (3 × 30 mL) and DMF (3 × 30 mL). The sample was left to be dried in an oven overnight at 85 °C [22].

#### Synthesis of perovskite

Nanoparticles of La_0.7_Sr_0.3_FeO_3_ perovskite were synthesized using citrate nitrate combustion technique. Typically, the synthesis method involved dissolving 0.73 g of cetyltrimethylammonium bromide (CTAB) in 15 mL of deionized water while stirring continuously to produce a homogenous solution. In 10 ml of deionized water, 0.61 g La (NO_3_)_3_·6H_2_O, 0.13 g Sr (NO_3_)_2_, and 0.82 g Fe (NO_3_)_3_·9H_2_O were mixed. Then, while continuously stirring, the nitrate aqueous solution was added to the CTAB solution. Thirty minutes of vigorous stirring were followed by the dropwise addition of 15 mL of NH_3_·H_2_O (25 wt% solution) into the combined solution. After that, the mixture was moved to a 50 mL Teflon-lined stainless autoclave. It was then tightly sealed and kept at 180 °C for nine hours. Following that, the autoclave was left to cool down on its own. Following the completion of the hydrothermal reaction, the brown precipitates were collected and repeatedly cleaned with ethanol and deionized water to get rid of any potential leftover ions. Following washing, the precipitates were dried for 24 h at 100 °C and collected. The as-synthesized sample was annealed at 700 °C for six hours in air to produce the final products [23].

### Characterization

The morphologies of the sorbents before and after adsorption were visualized using a Quanta FEG 250 (Czechoslovakia) field emission scanning electron microscope (FESEM) armed with energy-dispersive X-ray spectroscopy (EDX) systems, mag: 100, kV: 20, live time (s): 60, amp time (s): 3.84, take off: 45.4, resolution: (eV) 129.5. Fourier transform infrared spectra (FT-IR) of MOF and perovskite were measured using a Nicolet spectrophotometer Version 7.2 at the range from 500 cm^−1^ to 4000 cm^−1^. The crystalline phases of the samples were determined using PANalytical X-Ray Diffraction equipment model X’PertPRO with Cu-radiation (λ = 1.5424Å) at 45 kv, 35 mA and scanning speed 0.040 s^−1^ monochromator. The relative intensities and diffraction charts were obtained and compared with the corresponding ICDD files. The specific surface areas were measured using N_2_ adsorption–desorption isotherms (at 77.35 K) by using surface area analyzer (ASAP2000 Micrometric, UK) [22].

### Batch experiment studies

The adsorption isotherms were performed by placing solutions of MOF and perovskite with different initial concentrations (5-100 mg L^−1^) in a set of Erlenmeyer flasks (100 mL). Equal masses of CAF (0.01 g) were added to MOF and perovskite solutions and kept in an isothermal shaker at room temperature for 24 h to reach equilibrium of the solid-solution mixture. A similar procedure for another set of Erlenmeyer flask containing the same materials (MOF and Perovskite) concentration without CAF to be used as blank was implemented. The pH was adjusted by adding either few drops of diluted sodium hydroxide or sulphoric acid (0.1 mol L^−1^). Then, the flasks were removed from the shaker and the final concentration of materials in the solution was analyzed using spectrophotometer (UV-2600, Shimadzu). Then, the samples were filtered prior to analysis to minimize the interference of the CAF fines with the analysis. Each experiment was duplicated under identical conditions. Finally, the amount of adsorption at equilibrium, q_e_ (mg g^−1^), was calculated according to the following equation:

1 $${\mathrm q}_{\mathrm e}=\frac{\left(Co-Ce\right)v}w$$

Where v is the volume of the solution (L), C_o_ and C_e_ (mg L^−1^) are the liquid-phase concentrations of materials at initial and equilibrium, respectively, and w is the mass of dry adsorbent used (g).

The reusability of the adsorbents after CAF adsorption were carried out via mixing MOF (0.096 g) and perovskite (0.096 g) with (0.05 L) CAF at 20 mg L^−1^ at pH 3 for MOF and pH 7 for pervoskite. The materials have been washed with ethanol and water, dried, and reused.

### Quality assurance/quality control

The quality assurance and quality control have been assured via following the standard method of analysis during preparation, and characterization. The equilibrium was achieved during all the arrays except the contact time experiments. The adsorption measurements were triplicated and the mean values were taken in consideration. The error bars were implemented in all the adsorption experiments. The researchers have a relevant measure of qualifications and experience in material science, nanotechnology, and water treatment in order to demonstrate the competency when carrying out work of this nature, adding a further layer of confidence in the results produced.

## Analysis and discussion of the characterization data

### X-ray diffraction (XRD)

**Fig. 1:**
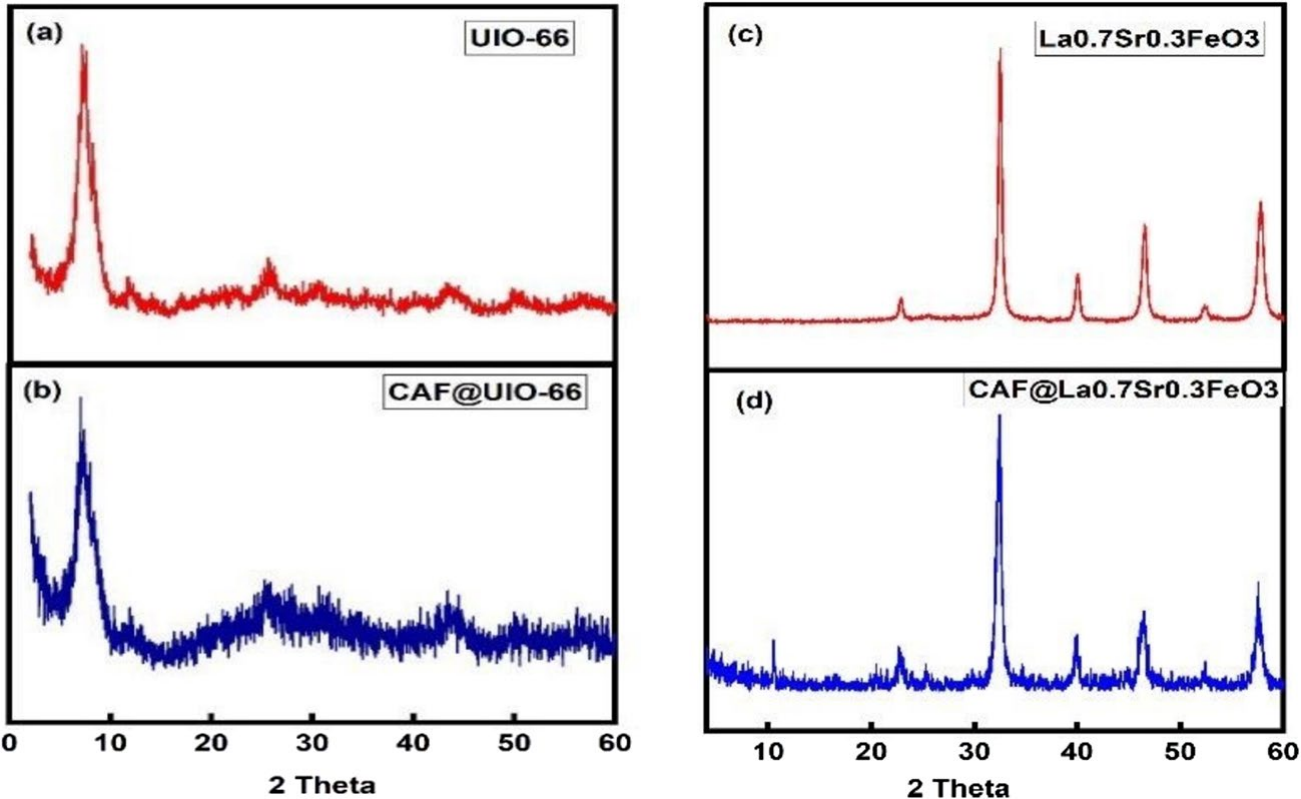
The XRD patterns of MOF (**a**, **b**) and perovskite (**c**, **d**) before and after CAF adsorption, respectively

The X-ray powder diffraction method was employed to detect the crystalline phases of the synthesized samples. The XRD patterns of MOF and perovskite before and after adsorption are plotted in Fig. 1. Figure 1a shows that there are three main peaks at 2θ = 7.2350^°^, 11.9150^°^ and 25.4150^°^ in the XRD spectrum of MOF before adsorption. The formation of the UIO-66 structure was confirmed by great agreement of the experimental XRD pattern with the theoretical powder pattern. The diffraction peak positions were found to be in agreement with the standard [24]. An unpronounced shift of all XRD peaks was observed which is related to the stability of the structure before and after adsorption process. The existence and/or absence of CAF on the active sites of MOF neither are detected in XRD pattern peak position nor intensities. This is according to the small concentration of the used material. However, the decrease in the intensity of the peaks suggests that the CAF is more likely to adsorb at the MOF inner surface.

The XRD patterns of perovskite (La_0.7_Sr_0.3_FeO_3_) before and after adsorption are plotted in Fig. 1c and d, respectively. The major phase coincides with the corresponding phase of orthorhombic perovskite structure of La_0.7_Sr_0.3_FeO_3_ with space group given by JCPDS 89-1269 [23]. It was observed that the peaks at 22.5087^◦^, 23.1229^◦^ and 54.1206^◦^ disappear after adsorption in addition to that the intensities of the other sharp peaks at (22.8362^◦^, 32.5005^◦^, 40.0754^◦^, 46.5042^◦^, 57.8059^◦^) have been lowered which suggest that the CAF is also adsorbed in the perovskite inner surface area. Moreover, the low intensity of XRD peaks after adsorption pointed to the interlayer penetration in addition to that superficially adsorbed. This could open new era of studies concerning the dose dependent crystal structure and the structural variations of (La_0.7_Sr_0.3_FeO_3_) before and after adsorption at different concentrations of CAF.

### Fourier transform infrared spectra (FT-IR)

**Fig. 2:**
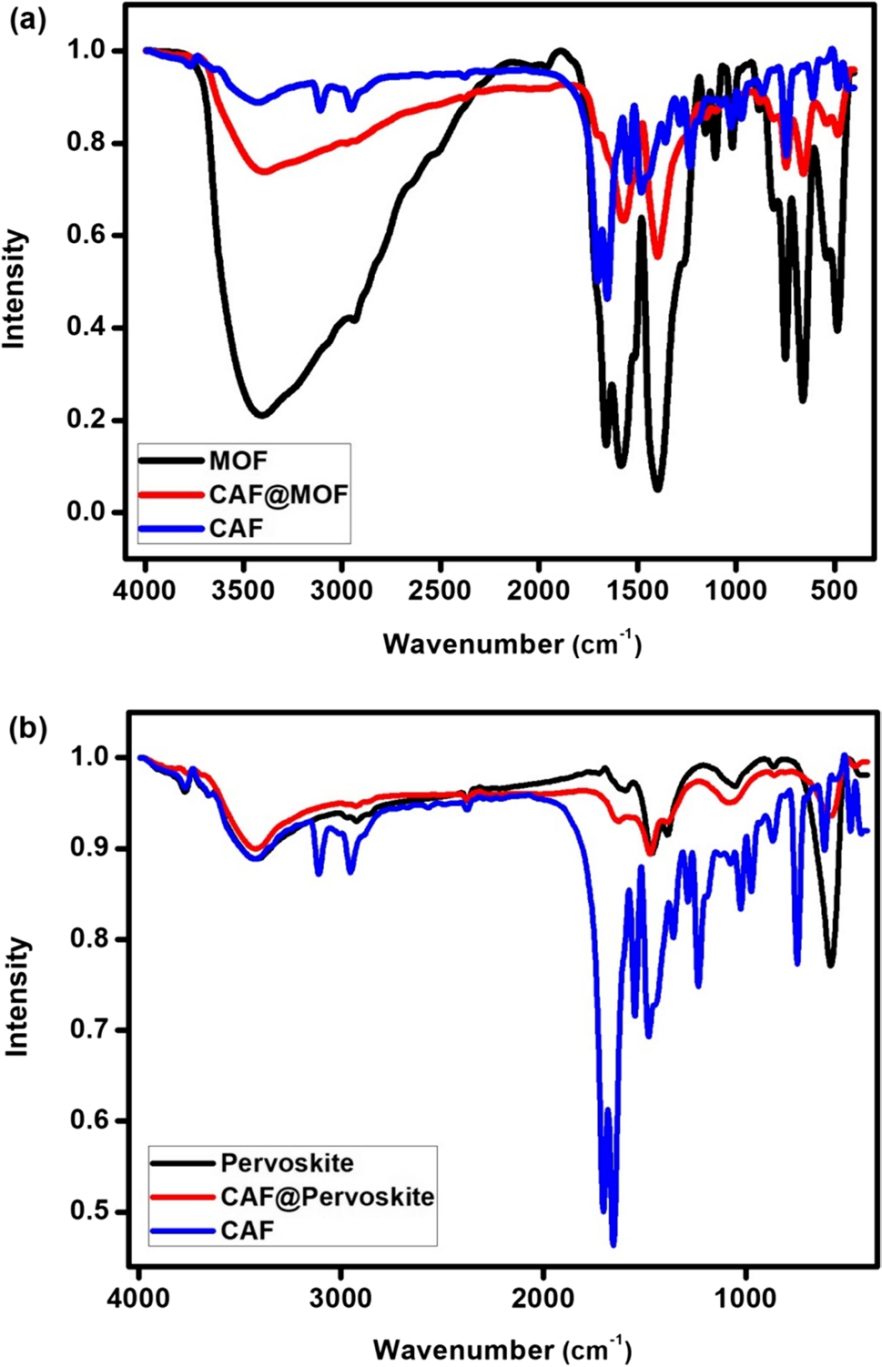
FTIR spectra of MOF (**a**) and perovskite (**b**) before and after CAF adsorption

FTIR spectrum of UiO-66 (Fig. 2a) shows strong absorption peaks at 1401 and 1601 cm^−1^. This is respectively attributed to the symmetric and asymmetric stretching vibrations of the carboxylate anion originated from 1,4-benzenedicarboxylic acid, which is the precursor of UiO-66. The vibrational band in the range of 3300 –2922 cm^−1^ represents C–H. A small band around 1512 cm^−1^ is corresponding to the vibration of C = C group in the benzene ring originated from 1,4-benzenedicarboxylic acid. The peak of C = O can be seen at 1712 cm^−1^. The peaks at 554 and 668 cm^−1^ represent Zr–O symmetric and asymmetric stretching, respectively. Appearance of new band after adsorption at 744 cm^−1^ is referring to CAF which confirms the adsorption of CAF onto UiO-66. Comparing between FTIR of MOF before and after adsorption one could also find that: the bands at 600–800 cm^−1^ and the band at 1500–1712 cm^−1^ are narrowed and the intensity of the band at 3000–3500 cm^−1^ is decreased. This could be explained by the new developed bonds of CAF@UiO-66.

The FTIR spectrum of perovskite (La_0.7_Sr_0.3_FeO_3_), Fig. 2b, shows a band at 558.7 cm^−1^ which is attributed to FeO stretching mode. The band at 856.3 cm^−1^ is well assigned to Sr–O stretching mode in 6-fold coordination. The bands at 1374.7 and 1452.3 cm^−1^ were well assigned to NO_3_ ^−1^ stretching mode. There is a peak of CAF which appears at 1475 cm^−1^ in the FTIR spectrum of the perovskite after CAF adsorption confirms the adsorption process. Comparing between FTIR spectra of perovskite before and after adsorption, it could be observed that the band at 510 cm^−1^ is decreased, and a new band was appeared after adsorption at 1475 cm^−1^ which refer to CAF. All of these changes confirm the adsorption of CAF onto perovskite.

### Field emission scanning electron microscope (FESEM)

**Fig. 3:**
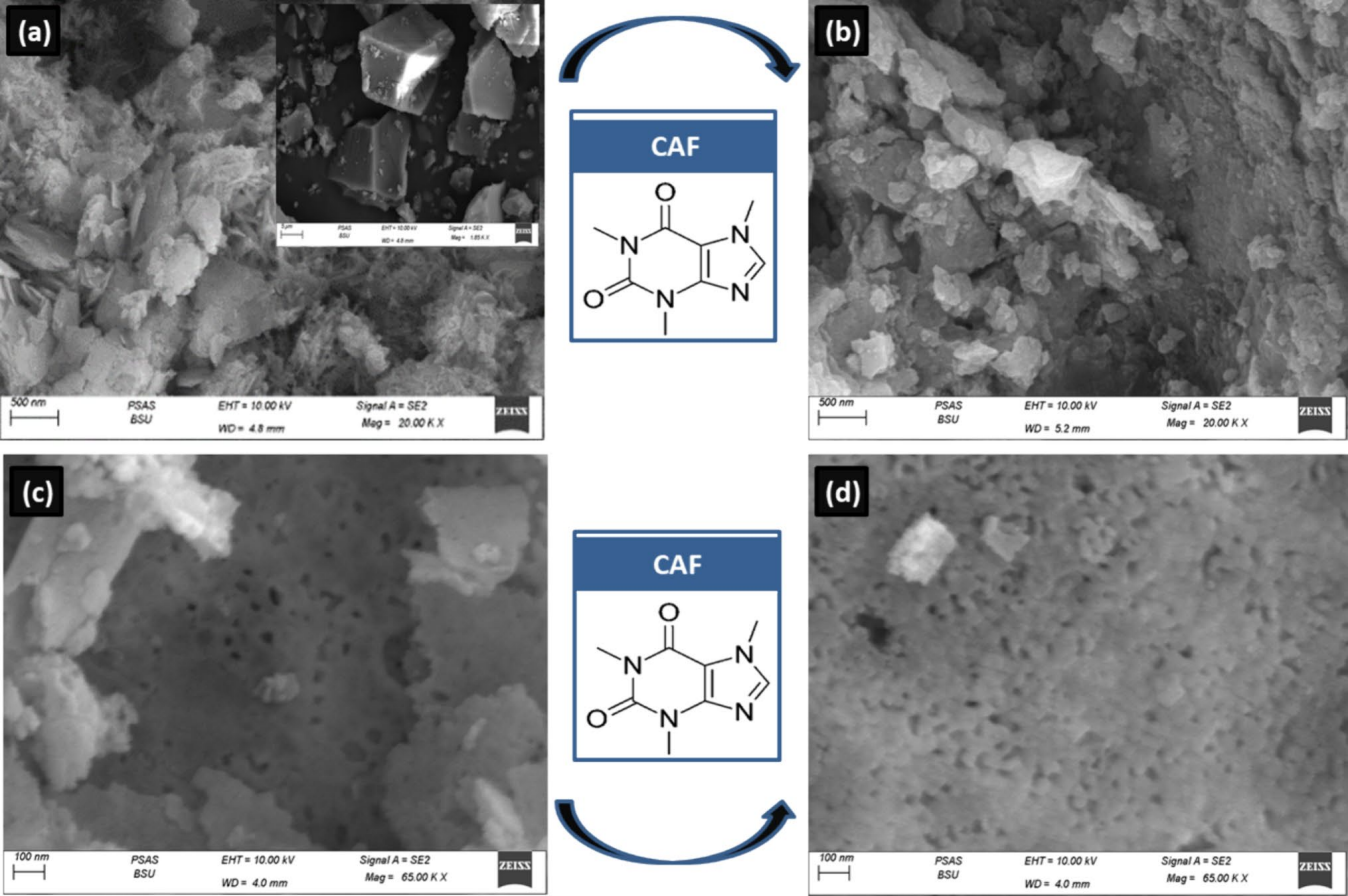

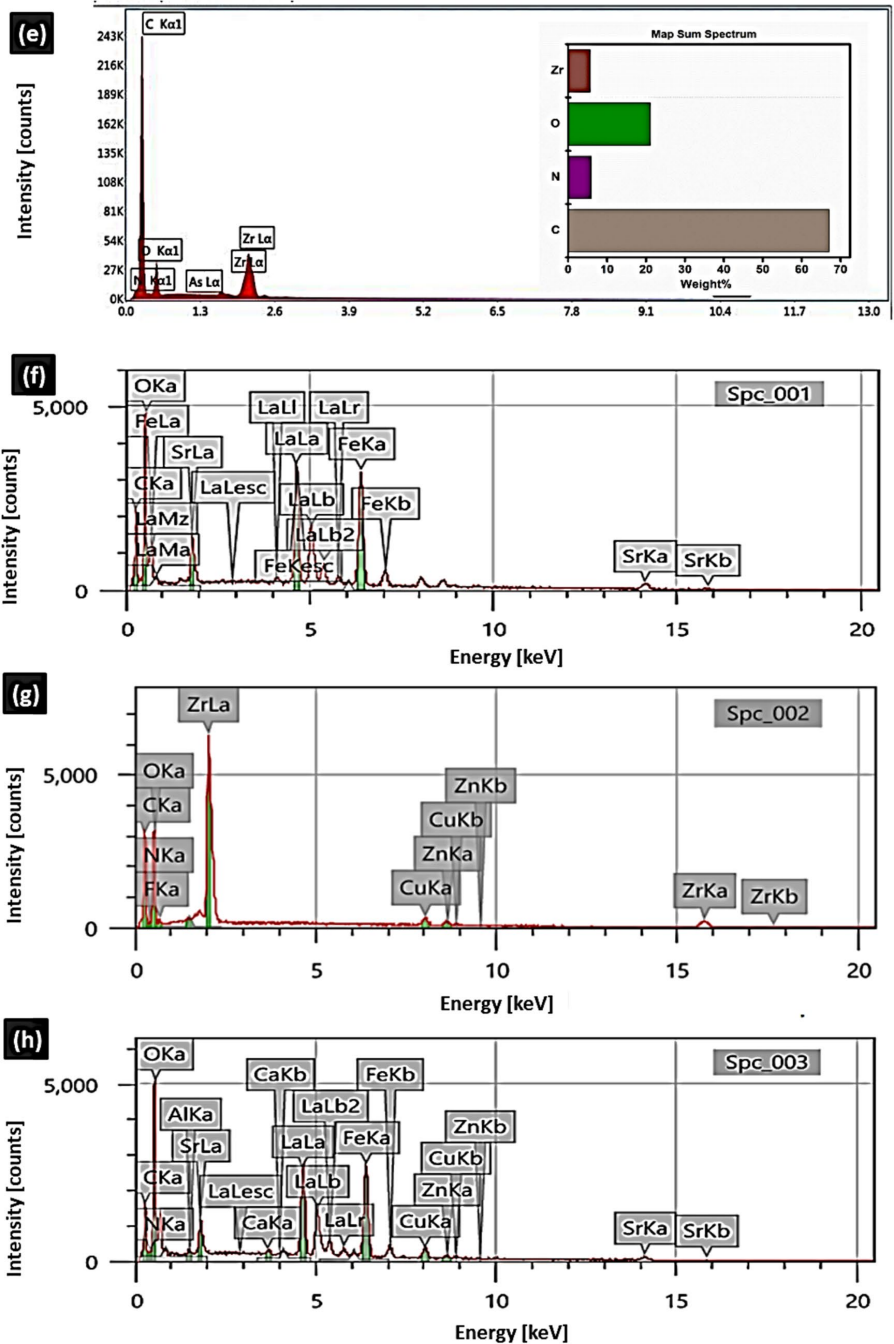
FESEM images of MOF before (**a**) and after (**b**) CAF adsorption and perovskite before (**c**), and after (**d**) CAF adsorption, and EDX spectra of MOF (**e**), perovskite (**f**), CAF@MOF (**g**) and CAF@ perovskite (**h**)

 The SEM image of MOF (Fig. 3a) shows octahedral crystals with sharp geometric boundaries and random orientation. There is no observation of other crystalline phase indicating the purity of the synthesized samples. After adsorption (Fig. 3b), the surface morphology seemed to be rougher with lower homogeneity and coalescence of the grains. This suggests that the accumulation of CAF onto the MOF occurred onto the outer surface of UIO-66. Figure (3c) shows that the developed perovskite has a porous structure. Smaller number of closed pores was found after adsorption of CAF onto perovskite (Fig. 3d) by comparison with those existing in perovskite (Fig. 3c). This is due to the filling of the surface-active site of the perovskite with CAF molecules. The adsorption of CAF onto La_0.7_Sr_0.3_FeO_3_ is occurred onto the outer surface area of the material. Moreover, the main trend appearing in the micrographs is the porous nature of the perovskite.

To know the synthetic structure of UiO-66 frameworks and perovskite before CAF adsorption, the energy dispersive X-Ray (EDX) spectrum of UiO-66 (Fig. 3e) shows multiple crystals of UiO-66 represents the spreading of metal Zr, C, O and N atoms throughout the crystal. The EDX spectrum of perovskite (Fig. 3f) represents the spreading of the metals La, Sr, Fe and O atoms throughout the sample. The EDX of the materilas after adsorption show the same constituents in addtion to to C, O, and N which ensure that adsorption of CAF onto MOF (Fig. 3g) and pervoskite (Fig. 3h).

### Surface area and pore characteristics

**Fig. 4:**
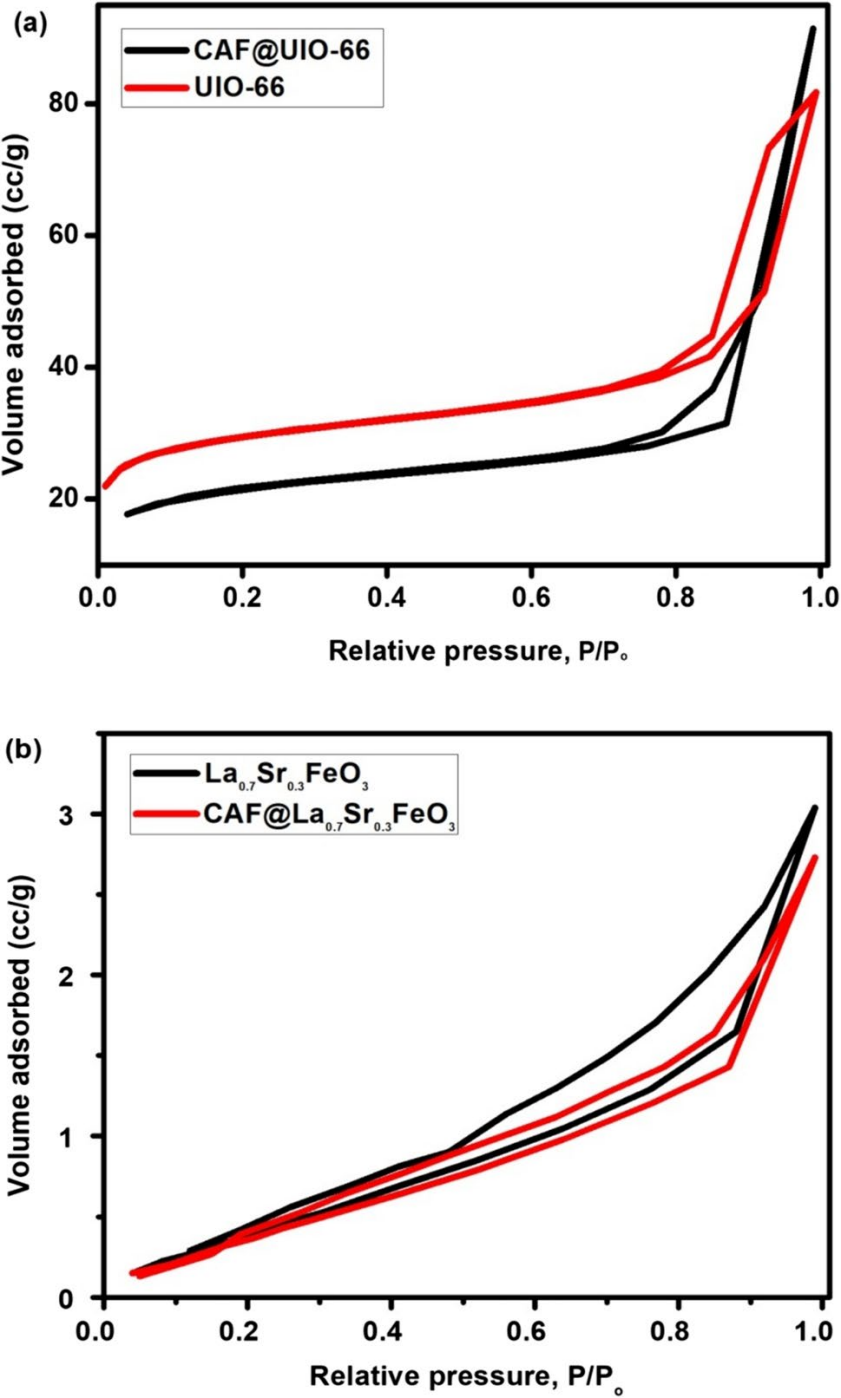
N_2_ adsorption/desorption isotherms at 77 K of CAF by MOF (**a**) and perovskite (**b**) before and after adsorption

Nitrogen adsorption–desorption isotherm studies were conducted to investigate the specific surface areas and porosities of UIO-66 and La_0.7_Sr_0.3_FeO_3_. The pore size distribution and the specific surface area of adsorbents were studied according to Barrett-jouner-Halenda (BJH) and Brunauer-Emmett-Teller (BET) methods, respectively (Fig. 4). According to IUPAC classification, the N_2_ isotherm of UiO-66 (Fig. 4a) is categorized as type IV with H_3_ type hysteresis loop, because of observing of a significant hysteresis loop in the relative pressure (P/P_o_) range of 0.5–1.0. The pore sizes of UIO-66 fall within mesoporous region (2–50 nm) [25]. The surface area and pore volume of UIO-66 is dropped from 651.67 m^2^ g^−1^ and 1.04 cm^3^ g^−1^ before adsorption to 468.60 m^2^ g^−1^ and 0.178 cm^3^ g^−1^ after adsorption, respectively. This suggested that the mechanism of CAF adsorption onto UIO-66 could be through the inner surface which agrees with the XRD results (Fig. 1b).

 Nitrogen adsorption/desorption isotherms of perovskite before and after adsorption at 77 K are shown in Fig. 4b. According to IUPAC classification, the isotherms can be classified as type IV with H_3_ type hysteresis loop. The inflection point of isotherms indicates the stage at which monolayer coverage is complete and multilayer adsorptions begin to happen [26].

The surface area and pore volume of perovskite is increased from 64.65 m^2^ g^−1^ and 0.135 cm^3^ g^−1^ before adsorption to 91.87 m^2^ g^−1^ and 0.199 cm^3^ g^−1^ after adsorption, respectively. This result is agreed with XRD results (Fig. 1b). The increment in surface area may be attributed to the presence of CAF group onto the surface of the perovskite which act as a new function group that attract nitrogen molecules. Another reason is the interlayer adsorption as suggested from XRD results.

## Analysis and discussion of the adsorption results

### Effect of pH

**Fig. 5:**
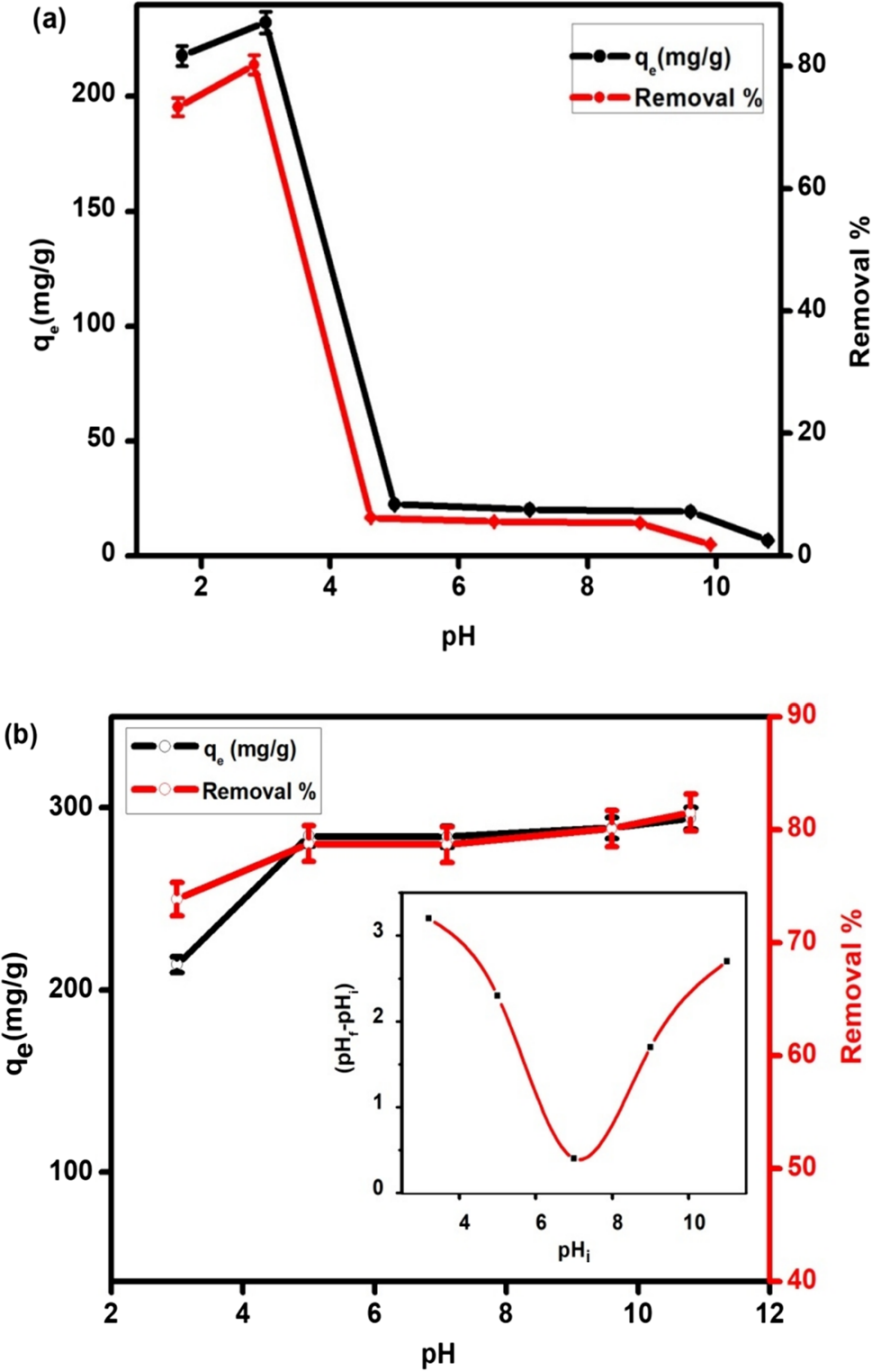
The effect of pH on the CAF adsorption onto MOF (**a**) and perovskite (**b**) at CAF initial conc. 30 mg L^−1^, v = 0.02 L and w = 0.01 g, with zeta potential charge of La_0.7_Sr_0.3_FeO_3_ (inset) (**b**)

It is well known that the pH affects the adsorption process because the degree of ionization of the adsorptive molecule as well as the surface charge of the adsorbent are affected by pH changes [27]. Different values of pH (1, 3, 5, 7, 9 and 11) have been used to be consistent with most of the tests for the same concentration. The results are displayed in Fig. 5. It is observed that the adsorption capacity of CAF by MOF increase in acidity. It is interesting to mention that point of zero charge (pH_ZPC_) for UiO-66 is 5.5 [28]. This means that the surface of the MOF has a positive charge below this value and a negative charge above this value, and the net charge will be negative in the neutral environment. Subsequently, the CAF adsorption capacity increased at lower values of pH (pH < 5.5) due to electrostatic attraction or binding with the induced positive charge of nitrogen atoms in CAF. On the other hand, the adsorption capacity in perovskite is stable in pH 4–10. It is important to mention that pH of isoelectric point (pH_iep_) for perovskite is 7.1, at this pH, both of adsorbent and adsorbate have no charge in suspension, but adsorption is very high, suggesting that some other factors plays roles in the adsorptive removal of CAF. The adsorption capacity of perovskite for CAF at pH 11 (strongly basic) is 20% less than by that at pH 7.1. Thus, the rest adsorption experiments were happened at pH 7.1 to elaborate the adsorption mechanism for neutral suspensions which simulate the normal conditions. Moreover, the results of pH show that perovskite can be used over a wide range of pH for CAF removal on a large scale which is considered as additional benefit of this sorbent towards CAF.

### Effect of initial concentration

**Fig. 6:**
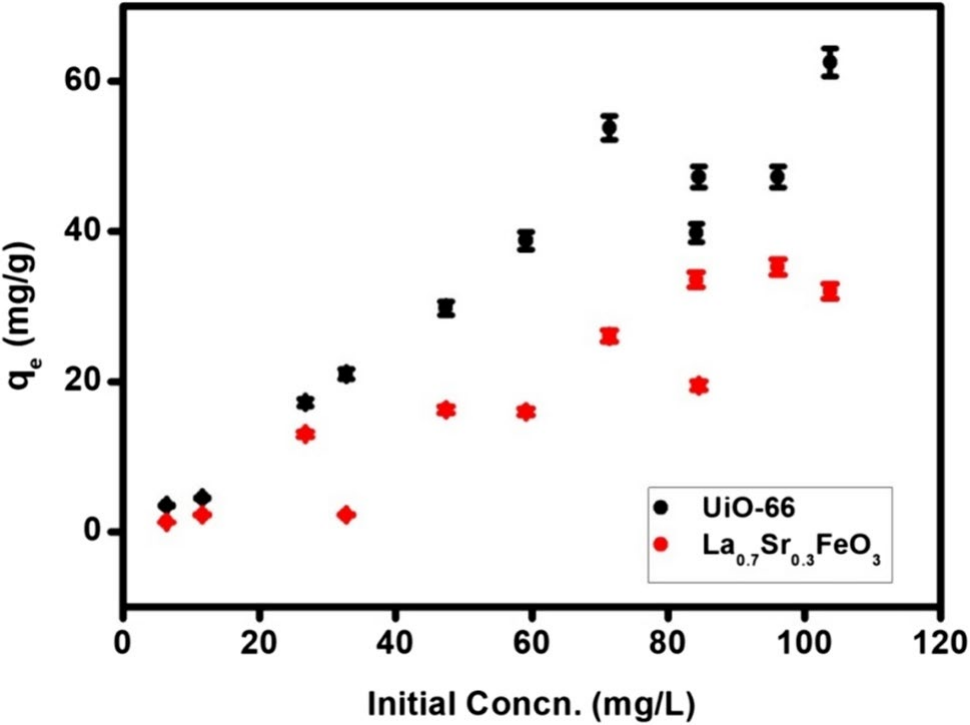
The effect of initial concentration of CAF on the adsorption capacities of MOF and perovskite at v = 0.02 L, w = 0.01 g, and pH 3 for MOF and pH 7 for perovskite

The adsorption process is also affected by CAF initial concentration. As the initial concentrations of CAF increased from 5 to 100 mg L^−1^, the adsorption capacity of UiO-66 toward CAF raised from 3.5 mg g^−1^ to 62.5 mg g^−1^ and for perovskite, it raised from 1.25 mg g^−1^ to 32 mg g^−1^ (Fig. 6). As can be seen, the adsorption capacity of CAF increased sharply with increasing the concentration of UiO-66 as well as perovskite at low initial concentration until reach equilibrium at higher concentration of CAF. This is probably due to the concentration gradient, which acts as the driving force to overcome the resistance of mass transfer of the organic compound from the aqueous phase to the solid one. Moreover, the higher initial concentration provides a higher driving force for this mass transfer thus leading to a higher adsorption capacity until saturation [19].

### Effect of contact time

**Fig. 7:**
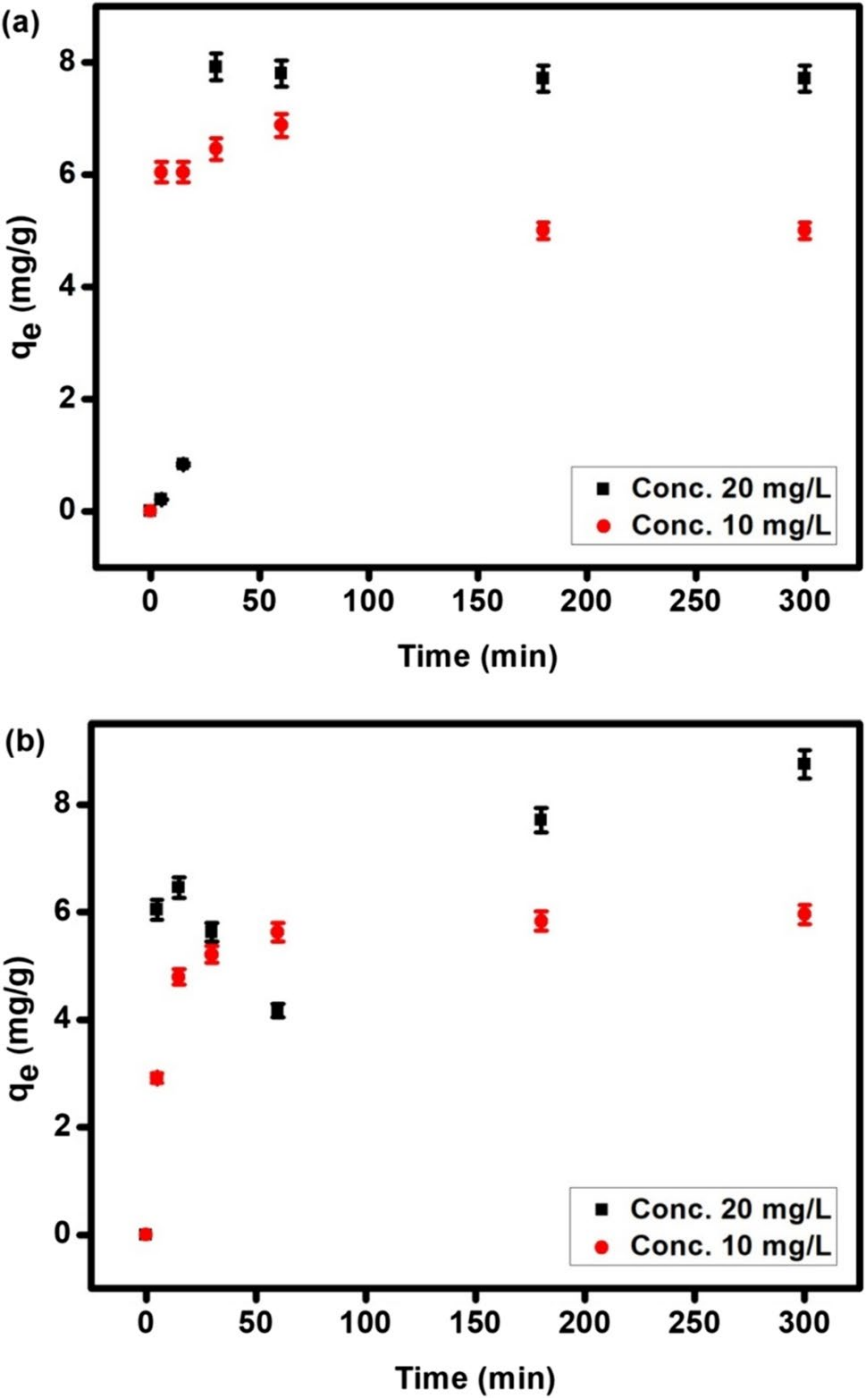
The effect of contact time of the adsorption CAF onto MOF (**a**) and pervoskite (**b**) at two different initial concentrations (10 and 20 mg L^−1^), w = 0.3 g, pH 3 for MOF and pH 7 for pervoskite and v = 0.5 L

One of the important parameters in the adsorption process is the contact time because it determines the rate of adsorbate removal. For MOF (Fig. 7a), the adsorption rate tends to be fast at the beginning of the adsorption process (the first 30 min). As the CAF molecules are in contact with free active sites onto MOF, with time, the adsorption rate becomes slower where the free active sites are lowered and the increase in the initial CAF concentration slightly cause decreasing in the adsorbed amount of CAF due to the rise of CAF molecules competing for the remaining available binding sites on the adsorbent. Thus, at higher times, the available active sites of the adsorbent become saturated. For perovskite, the adsorption capacity is increased very fast until the first 15 min, followed by a decrease in the rate up to 3 h until it reaches equilibrium where further increase in the time doesn’t impact the adsorptivity of the perovskite toward CAF.

### Adsorption kinetics

The adsorption kinetic shows the evolution of the adsorption process versus time. Numerous models were used to describe the diffusion of solutes at the surface and in the pores of adsorbents in batch adsorption systems. In this study, five models (pseudo-1st -order (PFO), pseudo-2nd -order (PSO), mixed 1st, 2nd-order, intraparticle diffusion and Avrami) have been studied in order to find the most appropriate model that describe the system. The kinetic is highly related to the initial solute concentration of the solution. However, PFO (Eq. 2) is desirable for higher initial concentration while PSO (Eq. 3) is preferable at lower concentration [27]. Pseudo-2nd -order supposes that the process is controlled by the adsorption reaction at the liquid/solid interface in the adsorbent [29]. This model is widely implemented to describe time evolution of adsorption under non equilibrium [30].

2 $${q}_{t}={q}_{e}(1-{e}^{-{K}_{1}t})$$

3 $${q}_{t}=\frac{{q}_{e}^{2} {k}_{2}t}{1+ {q}_{e} {k}_{2}t}$$

Where q_e_ (mg g^−1^) and q_t_ (mg g^−1^) refer to the equilibrium amount of CAF adsorbed and time-dependent amount of CAF adsorbed at time t (min), respectively. k_2_ (g mg^−1^h^−1^) represents the PSO rate constant and k_1_ (h^−1^) is the rate constant of PFO model [17].

Avrami model (Eq. 4) is the only model that shows “fractal-like behavior” (i.e., time-dependent adsorption rate coefficient) [31]. It is appeared that high approximation combined with the use of weight rather than volume fractions can lead to errors in the exponent (n) of as much as 0.3.

4 $${q}_{t=} {q}_{e}(1-\text{exp}{\left(-{k}_{av} t\right)}^{{n}_{av}})$$

Where n_av_ is the Avrami component (dimensionless) and k_av_ is the Avrami rate constant (min^−1^).

Many experimental systems may be described by the mixed 1st, 2nd-order model (Eq. 5). However, one cannot expect that such simple equations may describe behavior of any system consisting of e.g. polydisperse or structurally and energetically heterogeneous adsorbents. It shows that there might be more than one process running according to a simple exponential decay law. If the processes rate constants are much lower than that of the primary process, they may become visible, or if they are follow-up processes. The multi-exponential equation (m-exp) is simplest equation showing such properties, where the exponential terms corresponds to a certain part of the total equilibrium [32].

5 $${q}_{t}={q}_{e}\frac{1-\text{e}\text{x}\text{p}(-kt)}{1-{f}_{2}\text{exp}\left(-kt\right)}$$

Where K is the adsorption rate constant (mg g^−1^min^−1^) and f_2_ is the mixed 1, 2-order coefficient.

The intraparticle diffusion model describes the species transportation from the bulk to solid phase of porous material in solution as following:

6 $${q}_{t}={k}_{ip }\sqrt{t}+{c}_{ip}$$

Where K_ip_ is the measure of diffusion coefficient (mg g^−1^ min^−1(1/2)^) and c_ip_ is the intraparticle diffusion constant (mg g^−1^).

**Fig. 8:**
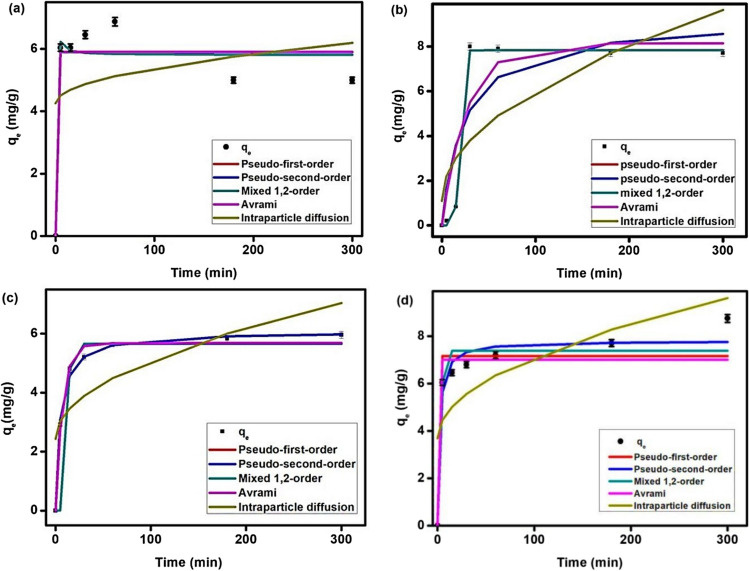
(**a**) Kinetic modelling of CAF onto MOF at concentration 10 mg L^−1^ (**a**a) and 20 mg L^−1^ (**b**) and onto perovskite at concentration 10 mg L^−1^ (**c**) and 20 mg L^−1^ (**d**), w = 0.3 g, pH 3 for MOF and pH 7 for pervoskite and v = 0.5 L

Figure 8 shows the fitting of the five kinetic models to the experimental data for the adsorption of CAF onto perovskite and MOF and the corresponding parameters are listed in Table 1.

**Table 1:** The parameters and correlation coefficients of the kinetic modeling of CAF adsorption onto MOF and perovskite

Model	Parameters	MOF (Conc., mg L^−1^)		Perovskite (Conc., mg L^−1^)	
		10	20	10	20
PFO	q_e_ [mg g^−1^]	5.90	8.14	5.68	7.15
	k_1_ [L mg^−1^]	5.41	0.03	0.13	9.84
	R^2^ [-]	0.91	0.83	0.99	0.9
PSO	q_e_ [mg g^−1^]	5.90	9.23	6.07	7.80
	k_2_ [g.mg^−1^h^−1^]	528.73	0.004	0.03	0.06
	R^2^ [-]	0.91	0.79	0.99	0.96
Mixed 1st, 2nd-order Model	q_e_ [mg g^−1^]	5.80	7.83	5.89	7.8
	K [mg.g^−1^ min^−1^]	0.0004	0.55	0.025	0.0004
	f_2_ [-]	1.0001	-33,503	0.87	0.99
	R^2^ [-]	0.91	0.99	0.99	0.96
Avrami	q_e_ [mg g^−1^]	5.90	8.14	5.68	7.06
	k_av_ [min^−1^]	2.11	0.13	0.8	0.93
	n_av_ [-]	1.81	0.28	0.16	0.41
	R^2^ [-]	0.91	0.83	0.99	0.92
Intraparticle diffusion	k_ip_ [mgg^−1^ min^(1/2)]^	0.11	0.49	0.26	0.34
	c_ip_ [mg g^−1^]	4.26	1.10	2.43	3.69
	R^2^ [-]	0.08	0.58	0.57	0.55

It can be seen from Fig. 8a and b and Table 1 that the mixed 1^s^ ^t^ 2nd-order model is the best one to describe the adsorption of CAF onto MOF at both concentrations under studies since the calculated adsorption capacities are close to the experimental one as well as the correlation coefficients (R^2^) are high as 0.91 and 0.99. Also, three models i.e., PFO, PSO and Avrami were presented with high correlation coefficients and the calculated and the experimental adsorption capacities are close. Additionally, these models are fitting the data at lower concentrations. On the other hands, it is clear that the intraparticle diffusion model is not appropriate for this system since R^2^ are low and the calculated adsorption capacities are differed than the measured one.

For perovskite, all the five models could describe the adsorption of CAF onto perovskite at the lower concentration while at the higher concentration, PSO and mixed 1, 2-order have been fitted the data better than Avrami and PFO. On the other hands, the intraparticle diffusion model is not appropriate for the adsorption of CAF onto perovskite where the correlation coefficients are low, and the calculated values of adsorption capacities didn’t match with the measured one.

### Adsorption equilibrium

Langmuir model (Eq. 7) supposes monolayer coverage of the adsorbate onto homogeneous sites on the adsorbent, without interactions between the adsorbed molecules.

7 $${q}_{e}={q}_{max}\frac{{K}_{L}c}{1+{K}_{L}c}$$

Where q_e_ is the amount adsorbed per unit mass of the MOF or perovskite (mg g^−1^) and q_max_ is the theoretical maximum adsorption capacity (mg g^−1^).

Freundlich model (Eq. 8) is used to appear on the adsorption process of heterogeneous surfaces [1].

8 $$\text{q}_\text{e}=\text{k}_\text{f}{C}_{e}^{1/n}$$

Where C_e_ is the equilibrium Conc. of CAF in the supernatant (mg L^−1^) [1]. Freundlich constant, K_f_, is describing the quantity of the adsorbates per gram of MOF or perovskite at the equilibrium concentration (L mg^−1^); n is used to measure the distribution of active sites related to the surface heterogeneity and indicates the strength and nature of the adsorption process [1].

Sips model (Eq. 9) does not assume a homogeneous surface or constant adsorption potential; it is more general than Langmuir model.

9 $$q_e=({Q}_{max}{K}_{s}{C}_{e}^{1/n})/(1+{K}_{s}{C}_{e}^{\frac{1}{n}})$$

Sips constant, k_S_, corresponding to the energy of adsorption; while value of (1/n) is close to 0, it describes heterogeneous adsorbent, and values close to 1 describe homogeneous distribution of binding sites [1].

A modified version of Langmuir is Langmuir-Freundlich model (Eq. 10).

10 $$q\text{e}= \frac{{q}_{MLF(K_{LF}{C}_{e})^{MLF}}}{1+{{(K}_{LF}{C}_{e})}^{MLF}}$$

Where 𝐾_LF_ is the constant for heterogeneous solid, q_MLF_ is Langmuir-Freundlich maximum adsorption capacity (mg g^−1^), and 𝑀_LF_ is the heterogeneous parameter.

Temkin isotherm supposes that adsorption sites on the adsorbent aren’t equal and have non similar adsorption coefficients and adsorption energies. According to Temkin model, due to the adsorbent –adsorbate interactions, the heat of adsorption decreases linearly with the surface coverage [1].

11 $${q}_{e}= \frac{RT}{{b}_{T}}ln({A}_T{C}_{e})$$

${b}_{T}$ is a constant related to heat of sorption (kJ mol^−1^), A_T_ refers to Temkin isotherm equilibrium binding constant (L.g).

Redlich–Peterson isotherm is an isotherm combining three parameters. It incorporates elements from both Langmuir and Freundlich equations, and the mechanism of adsorption does not follow ideal monolayer and it is a hybrid [33].

12 $${\mathrm q}_{\mathrm e}=\frac{AC_{e}}{1+BC_e^\beta}$$

Where B is a constant (L mg^−1^) ^β^, and β is an exponent that lies between 0 and 1. C_e_ is the concentration of equilibrium liquid-phase of the adsorbate (mg L^−1^).

Dubinin–Radushkevich (DR) equation is widely used to describe the adsorption in microporous materials, especially those of a carbonaceous origin. The equation is based on the assumptions of a change in the potential energy between the adsorbed phases and the gas and the characteristic energy of a given solid [34].

13 $$\mathrm W\:=\:\mathrm W0\;\exp\lbrack-\;\mathrm k(\mathrm e/\mathrm E)\sim\rbrack.$$

Where W/W_o_ is the part of adsorption space filled at relative pressure P/P_o_, e = RT in P_o_/P and k and ~ are constants) is used in its linear form.

Toth isotherm equation is a modulation of Langmuir equation to decrease the error between predicted values of equilibrium adsorption data and experimental data. The application of this equation is the best for the multilayer adsorption similar to BET isotherms which is a special type of Langmuir isotherm [33].

14 $${\Theta }=\frac{{K}_{T }p}{{[1+{{(K}_{T }p)}^{t}]}^{1/t}}$$

Where Θ = a/a_max_, a and a_max_ are the adsorption and the maximum adsorption, respectively, p is the equilibrium pressure and t and KT are the equation constants.

The Fritz–Schlunder isotherm equation (Eq. 15) is also investigated.

15 $${q}_{e}=\frac{{q}_{mFS }{k}_{FS}{c}_{e}}{1+{q}_{m}{c}_{e}^{mFS}}$$

Where K_FS_ Fritz–Schlunder equilibrium constant (L mg^−1^), q_mFS_ is Fritz–Schlunder maximum adsorption capacity (mg g^−1^), and m_FS_ is the Fritz–Schlunder model exponent.

**Fig. 9:**
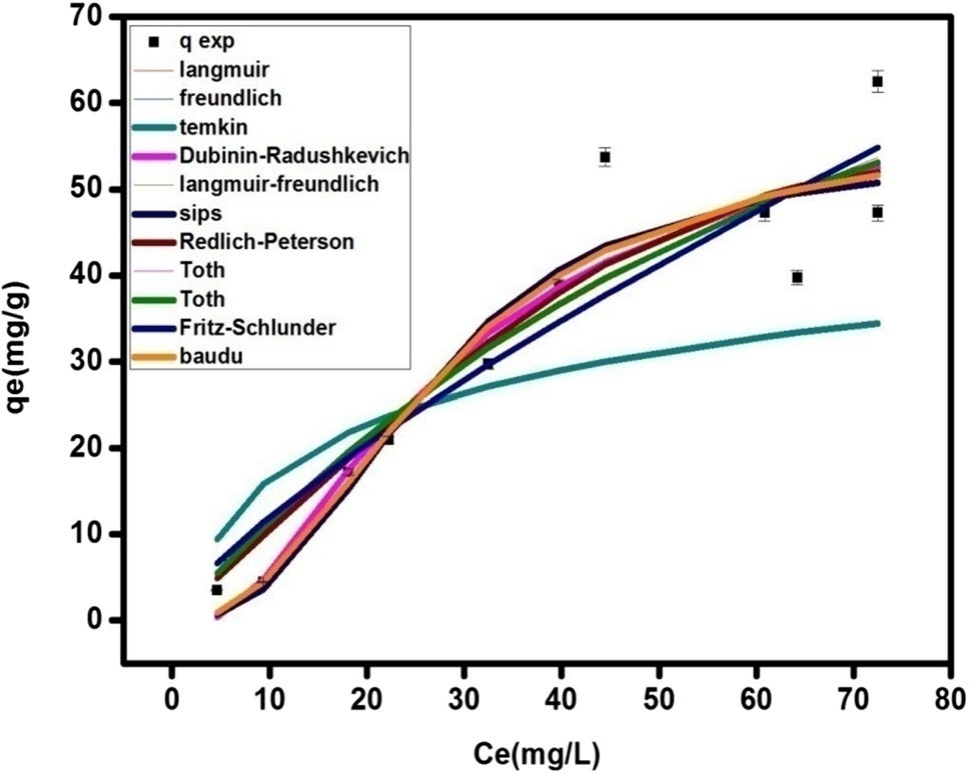
Adsorption isotherms modeling of CAF@MOF at different initial concentrations 5-100 mg L^−1^, w = 0.01 g, pH 3 and v = 0.02 L

Figure 9 and Table 2 show the results of the equilibrium adsorption isotherms modeling of CAF onto MOF. It can be seen that Sips and Langmuir-Freundlich models are the best to describe the fitting of the experimental data for the adsorption of CAF onto MOF, followed by Baudu model where the calculated values are close to the predicted one and R^2^ is very good (0.89). Khan and Langmuir model don’t describe the experimental data accurately. Although the correlation coefficient is good (R^2^ = 0.87 for both of them), however, the calculated values of the maximum adsorption capacity of MOF onto CAF is higher than the experimental one. On the other hands, Fritz-schlunder model resulted in lower adsorption capacity than the experimental one although R^2^ is acceptable, however, it also couldn’t fit the experimental data. Freundlich, Temkin, Toth and Redlich-Peterson models fit the data with acceptable values of R^2^ (0.85, 0.85, 0.87 and 0.88, respectively). The maximum adsorption capacity of CAF onto MOF is calculated using Dubinin-Radushkevich model equation and it yield higher value (q_m_ =76.54 mg g^−1^) while the correlation coefficient is high (R ^2^ = 0.89).

**Table 2:** The parameters of the adsorption models for CAF@UIO-66 and CAF@perovskite adsorption systems

Adsorption models		Parameter	MOF (UIO-66)	perovskite (La_0.7_Sr_0.3_FeO_3_)
Two- parameters isotherm	Langmuir	q_max_ [mg g^−1^]	129.40	1838.99
		K_L_ [L mg^−1^]	0.009	0.001
		R^2^ [-]	0.87	0.85
	Freundlich	K_f_ [L mg^−1^]	2.08	0.17
		1/n_F_ [-]	0.76	1.13
		R^2^ [-]	0.85	0.86
	Dubinin-Radushkevich	q_max_ [mg g^−1^]	76.54	64.03
		K_ad_ [mol^2^/kJ^2^]	0.005	0.014
		R^2^ [-]	0.89	0.84
	Temkin	b_T_ [kJ mol^−1^ ]	274.69	208.07
		A_T_ [-]	0.61	0.10
		R^2^ [-]	0.85	0.74
Three- parameters isotherm	Redlich-Peterson	K_R_ [L mg^−1^]	1.05	0.33
		a_R_ [-]	8.07E-06	0.04
		Β [L mg^−1^] ^β^	2.56	0
		R^2^ [-]	0.88	0.85
	Sips	q_m_ [mg g^−1^]	54.35	53.89
		Ks [L mg^−1^]	0.0002	0.0002
		1/n [-]	2.58	1.85
		R^2^ [-]	0.89	0.85
	Langmuir-Freundlich	𝑞_MLF_ [mg g^−1^]	57.14	53.53
		𝐾_LF_ [L mg^−1^]	0.03	0.01
		𝑀_LF_ [-]	2.28	2.08
		R^2^ [-]	0.89	0.94
	Toth	K_e_ [L mg^−1^]	1.40	0.002
		K_L_ [L mg^−1^]	0.004	0.04
		N [-]	1.15	0.006
		R^2^ [-]	0.87	0.85
	Kahn	q_m_ [mg g^−1^]	1355.31	95.80
		b_K_ [-]	0.0009	0.003
		a_K_ [-]	8.15	0
		R^2^ [-]	0.87	0.85
Four-parameters isotherm	Baudu	q_m_ [mg g^−1^]	49.90	49.73
		b_0_ [-]	0.0005	0.002
		X [-]	0.02	0.26
		Y [-]	1.30	5.16E-10
		R^2^ [-]	0.89	0.86
Five-parameters isotherm	Fritz-schlunder	q_mFSS_ [mg g^−1^]	16.98	16.83
		K_1_ [L mg^−1^]	0.13	0.03
		K_2_ [L mg^−1^]	0.12	1.95
		m_1_ [-]	0.75	1.15
		m_2_ [-]	0.10	0.026
		R^2^ [-]	0.85	0.86

**Fig. 10:**
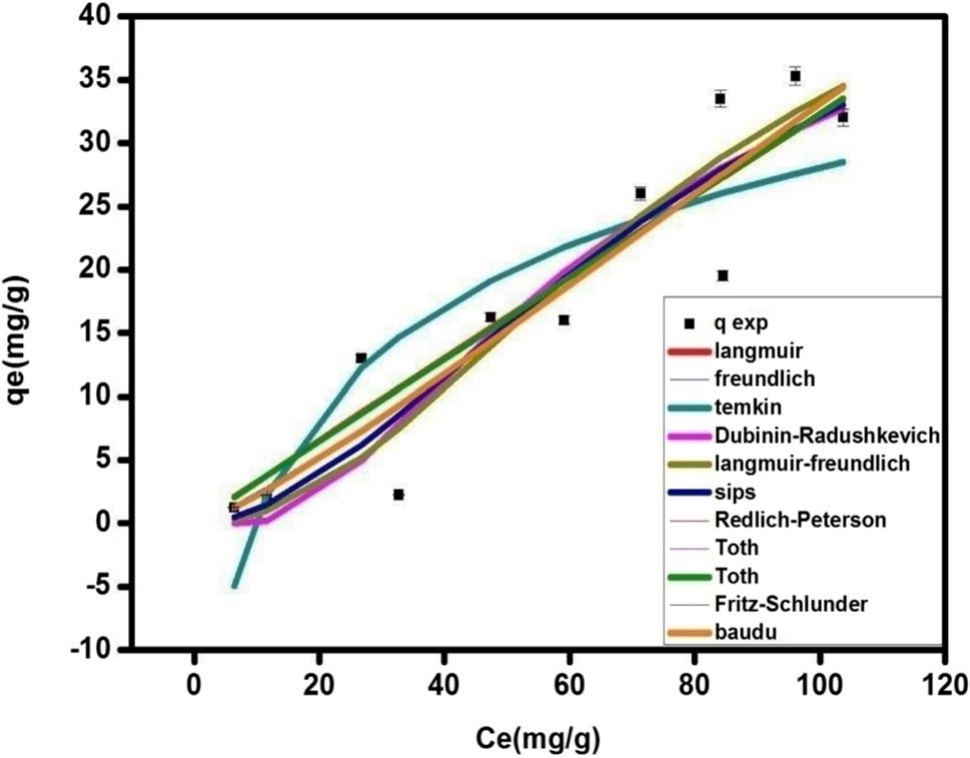
Adsorption isotherms modeling of CAF@perovskite at different initial concentrations 5-100 mg L^−1^, w = 0.01 g, pH 7 and v = 0.02 L

Concerning perovskite, the fitting of the nine adsorption isotherm models of CAF adsorption onto perovskite (Fig. 10; Table 2) show the equilibrium adsorption isotherms modeling of CAF onto La_0.7_Sr_0.3_FeO_3_. It can be seen that Baudu model is the best to fit the experimental data for the adsorption of CAF, (R^2^ = 0.86), followed by Sips and Langmuir-Freundlich models where the calculated values are close to the predicted one and the values of R^2^ are very good (0.85 and 0.94). Khan, Dubinin-Radushkevich and Langmuir model don’t describe the experimental data accurately. Although the correlation coefficients are good (R^2^ = 0.85, 0.84 and 0.85, respectively), however, the calculated values of the maximum adsorption capacity of CAF@La_0.7_Sr_0.3_FeO_3_ is higher than the experimental one. On the other hands, Fritz-schlunder model resulted in lower adsorption capacity than the experimental one while R^2^ is acceptable, however, it also couldn’t fit the experimental data. Freundlich, Toth and Redlich-Peterson models fit the data with acceptable values of R^2^ (0.86, 0.85, and 0.85, respectively). Temkin model yielded the lowest correlation coefficient (R^2^ = 0.74) which refers to that this model is not appropriate for the CAF@La_0.7_Sr_0.3_FeO_3_ system.

### Adsorption mechanism

The XRD analysis suggests that the pore filling and the interlayer penetration play a significant role in the adsorption of CAF onto MOF (Fig. 1b) and perovskite in addition to a superficial adsorption of CAF onto perovskite (Fig. 1d). This is also agreed with the SEM analysis where there are accumulations onto the surface of MOF and perovskite in addition to the pore filling in case of perovskite (Fig. 3b and d). The pore filling and the interlayer penetration of CAF onto MOF were also confirmed by the decrease in the specific surface area and pore volume after adsorption. The superficial adsorption of CAF onto perovskite is confirmed by the increase in the specific surface area after adsorption. According to the pH results, another mechanism is also contributed in the adsorption mechanism of CAF onto MOF; electrostatic attraction but it seems that this force is not significant for perovskite.

### Reusability of the adsorbents

**Fig. 11:**
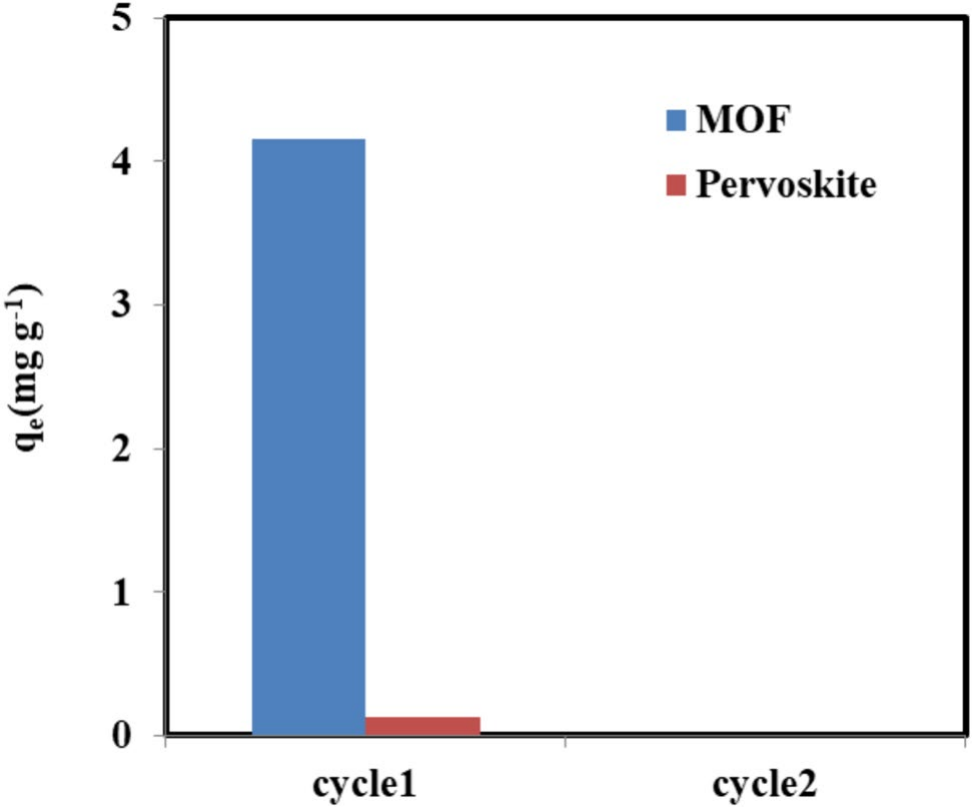
Multi-cycle adsorption of CAF onto MOF and perovskite at C_o_ 20 mg L^−1^, v 0.05 L, w = 0.096 g, pH 3 for MOF and pH 7 for pervoskite

The results of MOF and perovskite recycling after adsorption are represented in Fig. 11. It can be seen that the exhausted adsorbent has poor regeneration behavior since they were used for only one cycle. However, it is worthy to mention that CAF-loaded MIL-100 (Fe) MOF, was utilized as a smart corrosion-inhibiting coating to protect 308 L-16 stainless steel (308 L-16 SS) [35]. Additionally, CAF encapsulated in zirconium-MOFs have been introduced during the extrusion process to recycled polyamide 6 (PA6) and to a biopolymer based on polylactic acid (PLA) [36]. Hence, there is a possibility to investigate the exhausted adsorbents (CAF@MOF and CAF@perovskite) in different industrial applications which align with the principles of circular economy and zero-waste discharge. Also, waste-based adsorbents are recently investigated to manage various (organic/inorganic) emerging contaminates in wastewaters [37]. Subsequently, it is preferable to investigate the exhausted adsorbents CAF@MOF and CAF@perovskite to manage other pollutants. Moreover, further studies should focus on other emerging pollutants such as other PPCPs, microplastics, and perfluoroalkyl substances and it is highly recommended to investigate real wastewater samples.

### Strengths and limitations

This study deals with facile preparation methods of MOF and perovskite and investigating both materials for the removal of CAF as a model PPCP. Additionally, full adsorption isotherm and kinetic modeling were explored. This work also can be used as a guide for investigation of other PPCPs removal from water using the adsorbents under study. The characterization analysis in this study can be used to facilitate the modification of these adsorbents to improve their adsorptivity by functionalization and development of composite materials based on UIO-66 MOF and La_0.7_Sr_0.3_FeO_3_ perovskite. However still there are some limitations including: the high cost of the precursors used in the preparation of perovskite which restricts its production on the large scale. The chemical and mechanical stability of the developed MOF and perovskite materials were not investigated. Finally, we did not evaluate the developed materials in real wastewater treatment. Table 3 compares the finding of this study with the previous work of CAF adsorption onto other MOFs.

**Table 3:** A comparison of previous studies on the adsorption of CAF onto MOF

Type	Adsorbent	pH	Initial Conc.	Contact time	q_max_ (mg g^−1^)	Ref
MOF	UIO-66	N.A.	1–5 g·L^−1^	24 h	322	[3]
	UIO-66-COOH				267	
	Fe_3_O_4_@3DGA@ZIF-8	7.0	0.5 ∼ 30 mg/L	20 min	19.57	[38]
	UIO-66	3.5	5-100 mg L^−1^	2 h	62.5	This study My study
Perovskite	La_0.7_Sr_0.3_FeO_3_	4–10		3 h	35.25	

## Conclusion

UIO-66 MOF and La_0.7_Sr_0.3_FeO_3_ perovskite materials were synthesized successfully. Different characterization tests such as XRD, FESEM, FT-IR, and N_2_ adsorption–desorption isotherms were also carried out to identify the physico-chemical properties of the synthesized materials before and after adsorption. Then, the developed adsorbents have been used for the management of an emerging contaminant, CAF. The adsorption process was optimized via the main parameters. It was found that the maximum adsorption capacity of MOF (62.5 mg/g) is higher than that of perovskite (35.25 mg/g). This is attributed to the high surface area and pore volume of UIO-66 (651.67 m^2^ g^−1^ and 1.04 cm^3^ g^−1^, respectively) compared to perovskite (64.65 m^2^ g^−1^ and 0.135 cm^3^ g^−1,^ respectively) suggesting that the inner specific surface area of UIO-66 plays a significant role in the adsorption mechanism of CAF onto UIO-66. Additionally, the electrostatic attraction between CAF and UIO-66 enhances the overall adsorptivity compared to perovskite. Furthermore, different kinetics models were performed under optimized conditions and gave a better correlation with mixed 1st, 2nd-order for MOF and pseudo-2nd -order (PSO) and 1st, 2nd-order for perovskite. Based on our promising obtained results, MOF and perovskite are believed to be suitable and cost-efficient sorbents to manage CAF in water.

## Data Availability

All data listed or discussed during this work are included in this published article and its supplementary information files.

## References

[1] Gao Y, Liu K, Kang R, Xia J, Yu G, Deng S (2018). A comparative study of rigid and flexible MOFs for the adsorption of pharmaceuticals: kinetics, isotherms and mechanisms. J Hazard Mater.

[2] Yousefi M, Farzadkia M, Mahvi AH, Kermani M, Gholami M, Esrafili A (2024). Photocatalytic degradation of ciprofloxacin using a novel carbohydrate-based nanocomposite from aqueous solutions. Chemosphere.

[3] Sarker M, Jhung SH (2019). Zr-MOF with free carboxylic acid for storage and controlled release of caffeine. J Mol Liq.

[4] Olawale MD, Obaleye JO, Oladele EO (2020). Solvothermal synthesis and characterization of novel [Ni(ii)(tpy)(pydc)]·2H2O metal-organic framework as an adsorbent for the uptake of caffeine drug from aqueous solution. New J Chem.

[5] Beltrame KK, Cazetta AL, de Souza PSC, Spessato L, Silva TL, Almeida VC (2018). Adsorption of caffeine on mesoporous activated carbon fibers prepared from pineapple plant leaves. Ecotoxicol Environ Saf.

[6] Sacco O, Vaiano V, Matarangolo M (2018). ZnO supported on zeolite pellets as efficient catalytic system for the removal of caffeine by adsorption and photocatalysis. Sep Purif Technol.

[7] dos Santos Lins PV, Henrique DC, Ide AH, de Paiva e Silva Zanta CL, Meili L (2019). Evaluation of caffeine adsorption by MgAl-LDH/biochar composite. Environ Sci Pollut Res.

[8] Ribeiro Junior LA, Tromer RM, Dos Santos RM, Galvão DS (2021). On the adsorption mechanism of caffeine on MAPbI3perovskite surfaces: a combined UMC-DFT study. Phys Chem Chem Phys.

[9] Lee YR, Tian M, Kim SN, Ahn WS, Row KH (2014). Adsorption isotherms of caffeine and theophylline on metal-organic frameworks. Adsorpt Sci Technol.

[10] Li G, Wang T, Zhou S, Wang J, Lv H, Han M (2021). New highly luminescent 3D tb(III)-MOF as selective sensor for antibiotics. Inorg Chem Commun.

[11] Chavan SM, Shearer GC, Svelle S, Olsbye U, Bonino F, Ethiraj J, Lillerud KP, Bordiga S. Synthesis and characterization of aminefunctionalized mixed-ligand metal–organic frameworks of UiO-66 topology. Inorg Chem. 2014;53(18):9509–15. 10.1021/ic500607a. 10.1021/ic500607a 25148242

[12] Esrafili A, Ghambarian M, Yousefi M (2022). Electrospun zeolitic imidazolate framework-8/poly(lactic acid) nanofibers for pipette-tip micro-solid phase extraction of carbamate insecticides from environmental samples. Arab J Chem.

[13] Mazloomi S, Yousefi M, Nourmoradi H, Shams M (2019). Evaluation of phosphate removal from aqueous solution using metal organic framework; Isotherm, kinetic and thermodynamic study. J Environ Heal Sci Eng.

[14] Wang Y, He L, Li Y, Jing L, Wang J, Li X (2020). Ag NPs supported on the magnetic Al-MOF/PDA as nanocatalyst for the removal of organic pollutants in water. J Alloys Compd.

[15] Zhou T, Liang Q, Zhou X, Luo HJ, Chen W (2021). Enhanced removal of toxic hexavalent chromium from aqueous solution by magnetic Zr-MOF@polypyrrole: performance and mechanism. Environ Sci Pollut Res.

[16] Chen J, Ouyang J, Chen W, Zheng Z, Yang Z, Liu Z (2022). Fabrication and adsorption mechanism of chitosan/Zr-MOF (UiO-66) composite foams for efficient removal of ketoprofen from aqueous solution. Chem Eng J.

[17] Ouyang J, Chen J, Ma S, Xing X, Zhou L, Liu Z (2022). Adsorption removal of sulfamethoxazole from water using UiO-66 and UiO-66-BC composites. Particuology.

[18] Anastopoulos I, Pashalidis I, Orfanos AG, Manariotis ID, Tatarchuk T, Sellaoui L, et al. Removal of caffeine, nicotine and Amoxicillin from (waste)waters by various adsorbents. A review. J Environ Manage. 2020;261.10.1016/j.jenvman.2020.110236 10.1016/j.jenvman.2020.110236 32148306

[19] Gil A, Santamaría L, Korili SA (2018). Removal of Caffeine and Diclofenac from Aqueous Solution by Adsorption on Multiwalled Carbon nanotubes. Colloids Interface Sci Commun.

[20] Bilgin Simsek E, Tuna Ö, Balta Z (2020). Construction of stable perovskite-type LaFeO3 particles on polymeric resin with boosted photocatalytic Fenton-like decaffeination under solar irradiation. Sep Purif Technol.

[21] Wang Y, Zhu J, Zhang L, Yang X, Lu L, Wang X (2006). Preparation and characterization of perovskite LaFeO3 nanocrystals. Mater Lett.

[22] Abdelhamid HN (2020). UiO-66 as a catalyst for hydrogen production: Via the hydrolysis of sodium borohydride. Dalt Trans.

[23] Yao PJ, Wang J, Du HY, Qi JQ (2012). Synthesis, characterization and formaldehyde gas sensitivity of La 0.7Sr 0.3FeO 3 nanoparticles assembled nanowires. Mater Chem Phys.

[24] Zhao Q, Yuan W, Liang J, Li J (2013). Synthesis and hydrogen storage studies of metal-organic framework UiO-66. Int J Hydrogen Energy.

[25] Sing K (2001). The use of nitrogen adsorption for the characterisation of porous materials. Colloids Surf Physicochem Eng Asp.

[26] Rezlescu N, Rezlescu E, Dorin P, Doroftei C, Ignat M (2014). Composites: part B characterization and catalytic properties of some perovskites. Compos Part B.

[27] Bachmann SAL, Calvete T, Féris LA (2021). Caffeine removal from aqueous media by adsorption: an overview of adsorbents evolution and the kinetic, equilibrium and thermodynamic studies. Sci Total Environ.

[28] Azhar MR, Abid HR, Periasamy V, Sun H, Tade MO, Wang S (2017). Adsorptive removal of antibiotic sulfonamide by UiO-66 and ZIF-67 for wastewater treatment. J Colloid Interface Sci.

[29] Liu Y (2008). New insights into pseudo-second-order kinetic equation for adsorption. Colloids Surf Physicochem Eng Asp.

[30] Simonin JP (2016). On the comparison of pseudo-first order and pseudo-second order rate laws in the modeling of adsorption kinetics. Chem Eng J.

[31] Revellame ED, Fortela DL, Sharp W, Hernandez R, Zappi ME (2020). Adsorption kinetic modeling using pseudo-first order and pseudo-second order rate laws: a review. Clean Eng Technol.

[32] Marczewski AW (2010). Application of mixed order rate equations to adsorption of methylene blue on mesoporous carbons. Appl Surf Sci.

[33] Hamdaoui O, Naffrechoux E (2007). Modeling of adsorption isotherms of phenol and chlorophenols onto granular activated carbon: part II. Models with more than two parameters. J Hazard Mater.

[34] Nguyen C, Do DD (2001). The Dubinin-Radushkevich equation and the underlying microscopic adsorption description. Carbon N Y.

[35] Akhavan-Bahabadi Z, Zare HR, Mohammadpour Z (2023). Use of caffeine-containing MIL-100 (fe) metal organic framework as a high-performance smart anticorrosion coating to protect stainless steel in 3.5 wt% NaCl solution. J Coat Technol Res.

[36] Pina-Vidal C, Berned-Samatán V, Piera E, Caballero MÁ, Téllez C (2024). Mechanochemical Encapsulation of Caffeine in UiO-66 and UiO-66-NH2 to Obtain Polymeric composites by Extrusion with recycled polyamide 6 or Polylactic Acid Biopolymer. Polym (Basel).

[37] Suresh R, Rajendran S, Ponce LC. Waste-based adsorbents for the removal of emerging pollutants and their adsorption mechanisms. In: In Sustainable Technologies for Remediation of Emerging Pollutants from Aqueous Environment. Elsevier; 2024. p. 203–21. 10.1016/B978-0-443-18618-9.00024-3.

[38] Hua Y, Liu G, Lin Z, Jie Z, Zhao C, Han J (2024). Engineering of zeolitic imidazolate frameworks based on magnetic three-dimensional graphene as effective and reusable adsorbent to enhance the adsorption and removal of caffeine from tea samples. Food Chem.

